# A role for the nuclear receptor NR2F6 in peritoneal B cell homeostasis

**DOI:** 10.3389/fimmu.2022.845235

**Published:** 2022-08-16

**Authors:** William J. Olson, Bojana Jakic, Verena Labi, Johannes Woelk, Emmanuel Derudder, Gottfried Baier, Natascha Hermann-Kleiter

**Affiliations:** ^1^ Translational Cell Genetics, Department of Pharmacology and Genetics, Medical University of Innsbruck, Innsbruck, Austria; ^2^ Institute for Biomedical Aging Research, University of Innsbruck, Innsbruck, Austria; ^3^ Institute of Developmental Immunology, Biocenter, Medical University of Innsbruck, Innsbruck, Austria

**Keywords:** NR2F6 (nuclear receptor subfamily 2 group F member 6), B1 B cells, peritoneum, B cell survival, B cell migration

## Abstract

B cells are key mediators of humoral immunity. Mature B cells fall into various sub-classes that can be separated by their ontogeny, expression of cell surface markers, anatomical location, and function. B1 subsets play important roles in natural immunity and constitute the majority of B cells in newborns. In the adult, B1 cells predominate in the pleural and peritoneal cavities, while the mature B2 follicular subset makes up the major fraction of B cells in lymphoid tissue, although important subsets of antibody-secreting B1 cells are also present at these sites. B1 cells are the main producers of natural IgM but can also contribute to elimination of some pathogens, while B2 cells primarily mediate response to foreign antigens. The differential molecular underpinning of the B1 and B2 subsets remains incompletely understood. Here we demonstrate that germline-deficiency of the orphan nuclear receptor NR2F6 causes a partial loss of B1b and B2 B cells in the peritoneum while leaving peritoneal B1a cells unaltered. A competitive bone marrow chimera in *Nr2f6^+/+^* host mice produced similar numbers of *Nr2f6^+/+^* and *Nr2f6^-/-^* peritoneal B1b and B2 cells. The proliferation of *Nr2f6^-/-^* peritoneal B cells was not altered, while the migration marker CXCR5 was reduced on all subsets but Beta7-integrin was reduced only on peritoneal B1b and B2 cells. Similarly, B1b and B2 but not B1a cells, exhibited significantly reduced survival.

## Introduction

As the sole producers of antibodies, B cells are critical for defense against invading pathogens and response to vaccination but can also mediate humoral autoimmunity. Several subsets of mature B cells have been defined, including the innate-like B1a, B1b, and marginal zone B cells (MZB), as well as the B2 or follicular (FoB) subset. B1 cells are the primary producers of natural IgM, they secrete a large fraction of gut IgA and can rapidly generate antibodies in response to infection. For example, loss of B1 cells increases the susceptibility of mice to infection by *Streptococcus pneumonia* (1). On the other hand, follicular B2 cells are largely dependent on help from CD4^+^ T cells to produce antibodies and are ultimately responsible for the generation of long-lived high-affinity antibody-producing cells, but both B1 and B2 cells are known to participate in long-term protection from pathogens (2–4).

The earliest B1 cells appearing during ontogeny are B1a cells generated at embryonic day 9 from the yolk sac and the para-aortic splanchnopleura (5). Later during embryonic development, waves of B1a cells are generated from the fetal liver and neonatal bone marrow. Most B1b and B2 cell generation occurs in the neonatal and adult bone marrow (6–8). Work by the Herzenberg group has demonstrated that B1b and B2 cells can be generated from fetal and adult hematopoietic stem cells (HSC), whereas B1a were not reconstituted from either population, suggesting that B1a cells belong to a distinct lineage (9, 10). A recent work in preprint, using a Cas9 knock-in system to generate mice expressing a single immunoglobulin (Ig) pair, showed that when B cells expressed Ig found initially on a B1b cell, the Ig facilitated B1b and B2, but not B1a generation. Similarly, B2 Ig promoted both B1b and B2 differentiation, while B1a Ig primarily promoted the formation of B1a, again suggesting that B1a are a separate lineage from B1b and B2 (11). B1 cells predominate in pleural and peritoneal cavities in adult mice, while B2 cells make up the majority of B cells in other immune organs such as the spleen. In comparison, B1 cells constitute only about 2% of all B cells in the spleen (12). However, the functional characteristics of splenic and peritoneal B1 cells differ. For example, splenic B1 cells do not spontaneously secrete natural IgM *in vitro*, while peritoneal-derived B1 cells can do so (13). This differs from the *in vivo* situation in that both splenic and bone marrow resident B1 cells can produce antibodies, whereas peritoneal B1 cells do not (14). B1 cells from spleens express high levels of Notch and Notch targets such as *Mint-1*, while peritoneal B1 cells do not express these genes (13). The B2 cells populating the peritoneal cavity (PerC) can spontaneously secrete natural IgM *in vitro* and when transferred into severe combined immune deficient (SCID) mice, gain surface expression characteristics reminiscent of B1b cells (15). Environmental signals in the spleen versus the peritoneum are thought to contribute to the different characteristics of B1 cells at these locations (12). Both B1 and B2 cells from the peritoneum can migrate to other sites. For example, after activation, B1 cells in the peritoneum migrate to the spleen and lymph nodes to secrete antibodies (16–18).

Maintenance is especially critical for the B1 subset as they are largely produced during a brief window of fetal development and shortly after birth and thus must be maintained for the lifetime of the organism (19). B1a and to some extent B1b cells are thought to be maintained by slow-turnover predominately in the peritoneum (20). However, survival signals involved in B1 cell maintenance and homeostasis have not been well described. The B cell survival factor BAFF (BLyS) has been shown to promote survival of activated peritoneal B1 cells by downregulation of FcγRIIb but did not affect resting B1 cells (21). In accord with this data, studies in BAFF receptor-deficient mice have shown no significant impact on resting B1 cell numbers in the peritoneum (22). Whereas mature B2 survival in both spleens and lymph nodes has been linked to BAFF provided by T-zone reticular cells and fibroblastic reticular cells (23, 24). In contrast to peritoneal B1 cells, the survival of splenic B1 cells also appears to be BAFF-dependent (25). Work by two independent groups has linked secreted IgM to roles in BCR signaling in the context of development and turnover. Although, these studies come to different conclusions about the survival of peripheral B cells in the absence of secreted IgM (26, 27).

Residence and retention of B cells within the peritoneum is known to depend on a number of different receptors and integrins, Ansel and colleagues demonstrated a critical role for CXCR5 and its ligand CXCL13 in the homing and retention of B1 and B2 cells (28). Other groups have also described contributions of CXCR4, Beta7-integrin for migration to the PerC, Beta2-integrin for egress and Alpha4Beta1-integrins for both migration to and retention in the PerC (29, 30). The chemokine receptor CCR7 has also been shown to promote the egress of T and B cells from the PerC (31).

The nuclear receptor (NR) family can regulate immune responses and various members are known to promote or suppress inflammation in a context-dependent manner (32). Several NRs have been shown to participate in humoral immunity and autoimmunity *via* roles in both T cells and B cells (33). We have previously linked the orphan nuclear receptor NR2F6 (COUPTF-III, EAR2) to T cell function and defined it as an intracellular immune checkpoint in the context of cancer and as a suppressor of experimental autoimmune encephalomyelitis (EAE). Mice deficient for NR2F6 develop late-onset autoimmunity that resembles systemic lupus erythematosus. Our earlier work demonstrated a role for NR2F6 in the prevention of T-follicular helper (Tfh) cell accumulation after immunization, a phenomenon that likely contributes to the SLE-like disease observed in these mice by around 10 months of age (34–38). Mechanistically, the EAE and cancer effects are at least partly due to NR2F6 suppression of nuclear factor of activated T cells (NFAT) activity by competitive DNA binding (34, 39). In contrast, the role of NR2F6 in Tfh accumulation was associated with the suppression of IL-21 but did not appear to be due to interference with NFAT activity (37).

To date, NR2F6 has not been intrinsically linked to the B cell compartment and B1 B cells have not yet been investigated. In this report, we demonstrate that mice deficient for NR2F6 have lower numbers of B1b and B2 cells in the peritoneum. We find B2 cell numbers reduced in the omentum and mesenteric lymph nodes, and normal numbers in other tissues such as the spleen and inguinal lymph nodes. Migration markers CXCR5 and Beta7-integrin were mildly reduced on *Nr2f6^-/-^* peritoneal B1b and B2 cells. As measured by AnnexinV staining, apoptosis was significantly elevated in B1b and B2 cells isolated from the peritoneum of *Nr2f6^-/-^* mice, while B1a cells were unaffected. Our data, suggest that reduced B1b and B2 cells in the peritoneum of *Nr2f6*-deficient mice may be due to altered retention, lower survival or a combination of both factors.

## Materials and methods

### Mice

*Nr2f6*-deficient and -sufficient mice on the C57BL/6 background were used to characterize B cell populations (34, 39). Wild-type B6.SJL (CD45.1) mice on the C57BL/6 background were used as donors and recipients for adoptive transfer experiments. B6.SJL crossed to C57BL/6 (CD45.1/CD45.2) mice were used as hosts for the bone marrow chimera experiments, both recipients and donor mice in these experiments were 12-week-old males. For the remainder of the project, mice were used between the ages of 6-15 weeks, for each experiment, the mice were age-matched. No differences were seen in the peritoneal B cell populations based on gender therefore, the data presented here is derived from both male and female mice, although the majority of mice used were male. All animals were housed under specific pathogen-free conditions. Experiments were performed in accordance with Austrian guidelines (BMWFW-66.011/0064-WF/V/3b/2016; BMWF-66.011/0186-WF/V/3b/2016; BMWFW-66.011/0112-WF/V/3b/2017).

### Immune cell isolation

Total peritoneal cells were isolated by flushing the peritoneal cavity with 5ml of PBS containing 2% fetal calf serum (FACS buffer). Samples with visible blood contamination during the peritoneal lavage were excluded from further analysis. However, for consistency with other tissues investigated, such as the spleen, cells were suspended in erythrocyte lysis solution for five minutes at room temperature (R&D) and counted using the LUNA™ automated counter, dead cells were excluded by Propidium Iodide/Acridine Orange staining. Harvested peritoneal cells were resuspended in FACS buffer stained and analyzed by flow cytometry.

Spleens, a single inguinal lymph node (LN), all mesenteric LNs and equal numbers of Peyer’s patches (PP) per genotype were removed, spleen, LNs and PPs were crushed through a 100µm nylon mesh and the mesh washed with 5ml of complete IMDM medium. Bone marrow was isolated by opening both ends of one femur followed by flushing with 5ml FACS buffer with a needle and syringe. The omentum was isolated after peritoneal lavage, placed on a 100µm nylon mesh in a 6cm culture dish and manually disassociated using scissors. The mesh was then flushed with an additional 10ml of complete IMDM. Cells from spleen, lymph nodes and bone marrow were cleared of erythrocytes as described for the peritoneum above; cells were counted using the previously mentioned LUNA™ system. All samples were filtered again through a 40µm filter before staining and flow cytometry data acquisition (see below). Colons were removed and flushed with cold Hanks Balanced Salt Solution (HBSS), colons were then cut into small pieces (approx. 5mm) and incubated in disassociation buffer (HBSS with, 1mM DTT, 2mM EDTA) with shaking for 20min at 37°C. Colon pieces were then placed in digestion buffer (RPMI with 2.5µg/ml collagenase D, 1mg/ml DNase I, 2.5mM MgCl) followed by incubation on a shaker for 60 minutes at 37°C. Finally, colon pieces were pressed through a 100µM nylon mesh, rinsed with media and then filtered through a 40µM nylon mesh, the resulting suspension was counted and used for flow cytometry analysis.

### Adoptive cell transfer

Isolated splenocytes were counted, washed and suspended in PBS. 4x10^6^ cells were injected intra-peritoneally (i.p.) in a volume of 200µl. Harvest of transferred cells from the peritoneum was performed two days after adoptive transfer as described above. Cell counts were performed using the LUNA™ counter, also described above. Transferred cells were analyzed by flow cytometry.

### Flow cytometry

Single cell suspensions were incubated for 25 minutes at 4°C in FACS buffer (PBS+2% FCS). Antibodies used are as follows; CD5-FITC (53-7.3, BD), CD5-PerCPCy5.5 (53-7.3, Thermo Fisher), CD5-PeCy7 (53-7.3, BD), CD19-PeCy7 (6D5, BioLegend), CD11b-APC (M1/70, BioLegend), CD11b-Biotinylated (M1/70, BioLegend), CXCR5-Biotinylated (2G8, BD), Beta7 integrin-PE (FIB27, BioLegend), B220-PE (RA3-6B2, Thermo Fisher), CD45-bv500 (30F-11, BD), CD45.1-Pacific Blue (A20, BioLegend), CD45.1-APCCy7 (A20, BioLegend), CD45.2-FITC (104, BioLegend), CD45.2-PerCPCy5.5 (104, BioLegend), Ly6G-PE (1A8, BioLegend), F4/80-PECy7 (BM8, BioLegend), CXCR4-PerCPCy5.5 (LF26F12, BioLegend), B220-bv785 (RA3-6B2, BioLegend), CD19-bv510 (6D5, BioLegend), CD43-Fitc (S7, BD), CD23-bv510 (B3B4, BioLegend) and MHCII-bv421 (M5/114.15.2, BioLegend). After staining, cells were washed with 500µl of FACS buffer and analyzed. Stains requiring the addition of Streptavidin-APCCy7 (BioLegend) or APC (BioLegend) were incubated for an additional 20 minutes at 4°C, washed with 500µl of FACS buffer, and analyzed. For samples requiring AnnexinV staining, cells were washed and resuspended in AnnexinV staining buffer (Biolegend) containing pre-diluted AnnexinV-FITC (BioLegend). Intracellular Ki67-PE (BD) staining was performed using the Foxp3 staining kit available from eBiosciences and was done following the manufacturer’s instructions. Flow cytometry data was acquired using a FACSVerse (BD), FACSFortessa (BD), or FACSCanto II (BD). Data was analyzed using FlowJo software (BD).

### Bone marrow chimera

12-week old male recipient CD45.1/CD45.2 heterozygous *Nr2f6* ^+/+^ mice were lethally irradiated (1000 cGy). Total bone marrow from 12-week old male *Nr2f6^-/-^* (CD45.2) and *Nr2f6^+/+^* (CD45.1) mice was mixed at a 50:50 ratio and 2x10^6^ cells were transferred to irradiated recipient mice *via* i.v. injection. Host mice were maintained on neomycin (Sigma, N1876) delivered *via* drinking water for two weeks, neomycin was then removed and reconstitution was allowed to occur for an additional 10 weeks, for a total of 12 weeks. After reconstitution, total splenic and peritoneal cells were isolated and analyzed by flow cytometry as described above.

### Statistical analysis

Statistical analysis was performed using GraphPad Prism (version 8). Normality was determined using a Shapiro-Wilk test and for samples not normally distributed, significance was determined using a Mann-Whitney U test. Datasets displaying significance based on the Mann-Whitney U test are identified in the figure legends. For the remaining, normally distributed data, a two-tailed student t-test was used to determine significance. P-values derived from both the student t-test and Mann-Whitney U test were considered significantly different as follows; *<0.05, **<0.01, ***<0.001 and ****<0.0001.

## Results

### Peritoneal B1b and B2 cell numbers are reduced in *Nr2f6*-deficient mice

**Figure 1:**
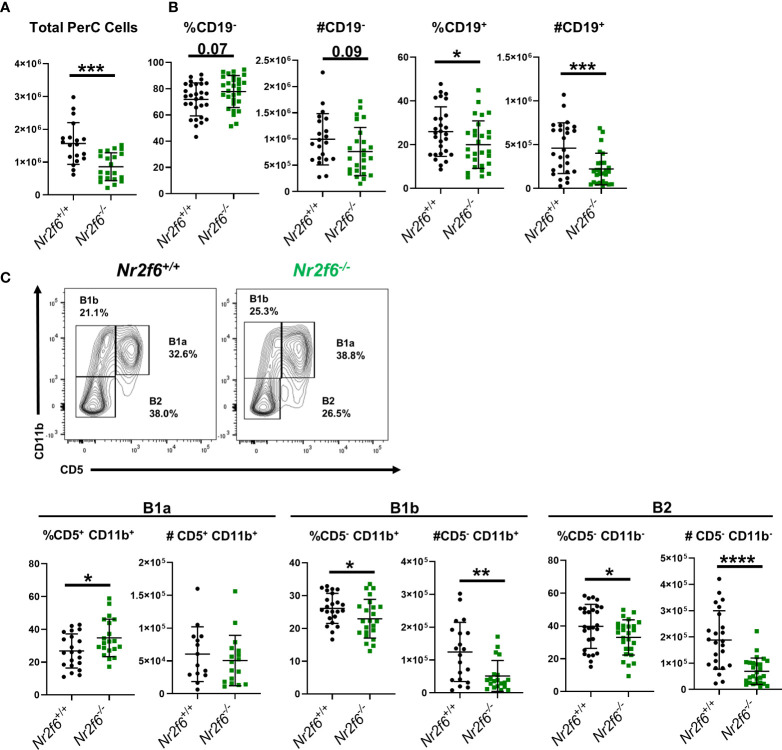
Germ-line *Nr2f6*-deficiency results in lower B1b and B2 cell numbers in the peritoneum. **(A)** Total peritoneal cells were enumerated after peritoneal lavage from *Nr2f6^+/+^* and *Nr2f6^-/-^* mice. **(B)** Percentage and total numbers of CD19^+^ and CD19^-^ cells were determined by flow cytometry. **(C)** B1a, B1b and B2 cell populations were further defined by flow cytometry using the markers CD5 and CD11b. Flow data was used to calculate total numbers of each B cell population. Each data point represents an individual mouse, data are from at least five individual experiments. For most data sets statistical significance was determined using a two-tailed student t-test however, all data was tested for normality by Shapiro-Wilk testing, and for non-normally distributed data, significance was determined using a Mann-Whitney U test. Within this figure, **(B)** total cell counts for CD19^+^, CD19^-^ and **(C)** total cell counts for B1a, B1b and B2 were not normally distributed and thus tested by a Mann-Whitney U test, significant p-values for all tests are displayed as *<0.05, **<0.01, ***<0.001 and ****<0.0001.

**Figure 2:**
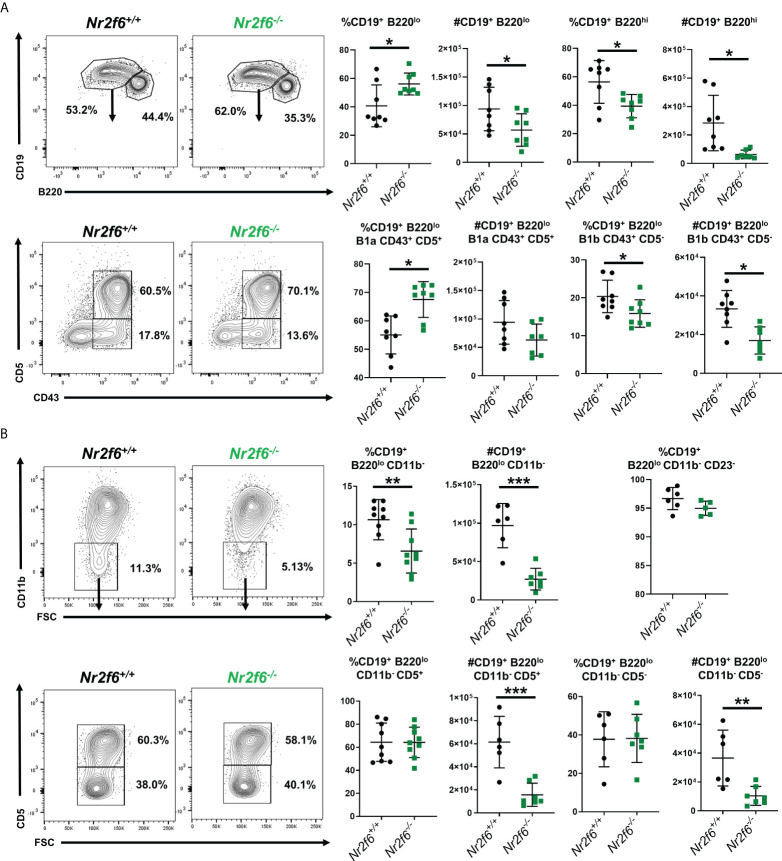
Further definition of B1 and B2 cells in the peritoneum of *Nr2f6^-/-^* mice. **(A)** Total peritoneal B1 cells (CD19^+^ B220^lo^) and B2 (CD19^+^ B220^hi^) were investigated by flow cytometry, and representative FACS plots are shown. A summary of the frequency and total counts for total B1 cells are shown on the right. B1a and B1b cells were further defined from the CD19^+^ B220^lo^ population using CD43 and CD5, a representative FACS plot as well as frequency and counts from all mice investigated are shown on the right (lower row). **(B)** Characterization of CD11b^-^ cells within the total CD19^+^ B220^lo^ population, representative FACS plots are shown as well as a summary of all mice investigated. Frequency and total cell counts of CD19^+^ B220^lo^ CD11b^-^ are shown in the upper row, staining for CD5^+^ cells are shown in the lower row. The frequency of CD23^-^ cells is also shown from the CD19^+^ B220^lo^ gate (upper right panel). Each data point represents an individual mouse, data shown are from at least two individual experiments with at least two mice of each genotype per experiment. Statistical significance was determined using a two-tailed student t-test significant p-values are displayed as *<0.05, **<0.01 and ***<0.001.

We have previously demonstrated that young *Nr2f6*-deficient mice have normal numbers of immature T1 B cells, MZB and FoB cells in the spleen, however, B1 cells were not analyzed (34). Here, we investigated in greater detail whether global NR2F6-loss affects B1 and B2 cells or their homeostasis. Because the peritoneum is a major site for B1 cell activation and long-term residence, we first assessed peritoneal B cell populations from *Nr2f6^+/+^* and *Nr2f6^-/-^* mice *via* flow cytometry. In the absence of NR2F6, total peritoneal cell numbers were significantly reduced (**Figure 1A**). Despite a trend toward fewer cells, CD19^-^ non-B cells did not contribute significantly to this alteration, but both the frequency and total counts of CD19^+^ cells were reduced in *Nr2f6^-/-^* mice (**Figure 1B**). Notably, the fraction of B1a (CD19^+^, CD11b^+^, CD5^+^) cells was elevated, whereas the total numbers of B1a cells were unchanged, B1b (CD19^+^ CD11b^+^ CD5^-^) and B2 cell (CD19^+^ CD11b^-^ CD5^-^) fractions and total numbers were significantly reduced in the peritoneal lavage of *Nr2f6^-/-^* mice (**Figure 1C**). These results were confirmed using an alternative gating strategy, defining the B1a, B1b and B2 cells using CD19, B220, CD43 and CD5 (**Figure 2A**). Several studies have demonstrated a population of B1 cells that do not display CD11b (40, 41). Therefore, we investigated this population to determine if they were also reduced in the peritoneum of *Nr2f6*-deficient mice. The CD11b^-^ population was quantified from total CD19^+^ B220^lo^ cells and we found that these cells were nearly all CD23^-^ (**Figure 2B**, top right panel). A significant reduction in both frequency and total cell counts of CD11b^-^ was observed in the *Nr2f6*-deficient peritoneum (**Figure 2B**). The fraction of CD19^+^ B220^lo^ CD11b^-^ cells displaying CD5 was unaltered in the absence of NR2F6, although total cell counts were significantly decreased due to the reduced CD11b^-^ fraction (**Figure 2B**). The composition of the CD19^-^ population was further investigated by flow cytometry to determine if *Nr2f6*-deficiency resulted in alterations to any non-B cell populations. However, no changes could be observed in total T cell (CD45^+^, CD3^+^) (**Supplementary Figure 1A**), eosinophil (CD45^+^, SSC^hi^, Ly6G^lo^), large peritoneal macrophage (CD45^+^, F4/80^hi^, MHCII^lo^) or small peritoneal macrophage (CD45^+^, F4/80^lo^, MHCII^hi^) numbers (**Supplementary Figure 1B**) (42, 43). Neutrophils (CD45^+^, CD11b^+^, Ly6G^+^) were mildly but not significantly reduced in *Nr2f6*-deficient mice. Within the monocyte population, non-inflammatory monocytes (CD45^+^, CD11b^int/lo^, Ly6G^-^, CD115^+^, Ly6C^lo^) trended higher but were not significantly different, inflammatory monocytes (CD45^+^, CD11b^int/lo^, Ly6G^-^, CD115^+^, Ly6C^hi^) tended to lower numbers in the absence of NR2F6 but also did not reach significance (**Supplementary Figure 1B**). We further confirmed that the gating strategy used to define the monocyte populations excluded small peritoneal macrophages (**Supplementary Figure 1C**). Together, our data indicate that total peritoneal cell numbers are reduced in *Nr2f6^-/-^* mice and that this is largely due to reductions in the B1b and B2 B cell populations.

### B cell numbers are reduced in the mesenteric lymph node and omentum of *Nr2f6* ^-/-^ mice

**Figure 3:**
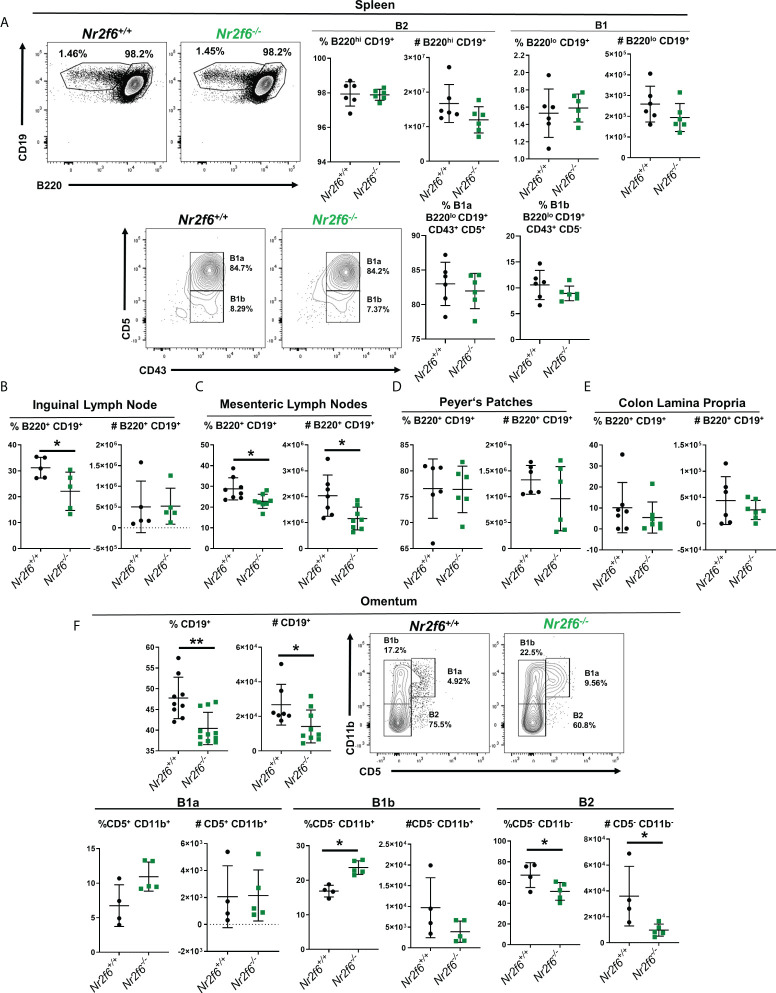
B cells are reduced in the mesenteric lymph nodes and omentum in the absence of NR2F6. Flow cytometry analysis of B cells at multiple locations of *Nr2f6*-sufficient and –deficient mice. **(A)** B1 and B2 populations in the spleen were determined by labeling of B220 and CD19 as indicated. Cell numbers were calculated from the flow cytometry data. B1a and B1b cells were further defined by CD43 and CD5 expression (lower row). **(B, C)** B cells in indicated lymph nodes were stained for B220 and CD19 and frequency was used to determine total B2 cell numbers. Lymph node data is based on a single inguinal lymph node, while mesenteric lymph node numbers are derived from the total mesenteric lymph nodes in each mouse. **(D)** Peyer’s patches were removed from the small intestine a total of six were pooled per mouse and stained for CD19 and B220 to determine B cell numbers. **(E)** Colons were disgested and filtered and B cells were identified by CD19 and B220 staining. **(F)** Lymphocytes were isolated from the ommentum by physical disruption and total B cells were enumerated by CD19 staining. B1a, B1b and B2 cells were defined from CD19^+^ cells by CD11b and CD5 staining (lower row). Each data point represents an individual mouse, data shown are from at least two individual experiments with at least two mice of each genotype per experiment. For most data sets statistical significance was determined using a two-tailed student t-test however, all data were tested for normality by Shapiro-Wilk testing, and for non-normally distributed data, significance was determined using a Mann-Whitney U test. Within this figure, **(B)** total CD19^+^ B220^+^ and **(F)** CD19% in the omentum were not normally distributed and were tested for significance by a Mann-Whitney U test, significant p-values for all tests are displayed as *<0.05 and **<0.01.

In our previous work, we had not fully defined the B cell populations in the spleen or other sites, therefore we next asked if B1 or B2 cell numbers were generally reduced in other tissues or if this phenomenon was relegated to the peritoneum. Spleens were investigated first as it contains both B1 and B2 cells in the steady-state and B1 and B2 cells are known to migrate to this site from the peritoneum (16, 18). B cell populations were defined by flow cytometry with B1 cells, designated as CD19^+^ B220^lo^ and B2 as CD19^+^ B220^hi^. No differences in total B1 or B2 populations by either frequency or cell number were found in spleens of *Nr2f6*-deficient mice relative to controls (**Figure 3A**). The CD19^+^ B220^lo^ B1 population was further defined by CD43 and CD5 staining, and no differences were observed in the fraction of cells staining as B1a (CD43^+^, CD5^+^) or B1b (CD43^+^, CD5^-^) in *Nr2f6*-deficient spleens relative to controls (**Figure 3A**, lower row). We then investigated other sites known to contain B cells, emphasizing tissues associated with the peritoneum. Inguinal lymph node B2 cell (CD19^+^, B220^hi^) frequency was significantly reduced, but total cell numbers were not affected (**Figure 3B**). While in the mesenteric lymph node, significant reductions in both B2 cell (CD19^+^ B220^hi^) frequency and cell number were observed in *Nr2f6*-deficient mice relative to *Nr2f6*-sufficient mice (**Figure 3C**). Of note, in both the inguinal and mesenteric lymph nodes, B1 (CD19^+^ B220^lo^) cells were not found in abundance (**Supplementary Figure 2** and data not shown). Another common enteric site for B2 cells are the Peyer’s patches, however, B2 cells were found at similar frequency and cell number between the genotypes (**Figure 3D**). B cells have been shown to migrate directly from the peritoneum into the colon lamina propria therefore we investigated this site for B cells in the context of *Nr2f6*-deficiency (44). Within the colonic lamina propria, B2 (CD19^+^ B220^hi^) cells were similar by both frequency and numbers in the absence of NR2F6 (**Figure 3E**). Berberich and colleagues previously demonstrated that peritoneal B2 cells not attached to the local matrix of the peritoneum could migrate into the omentum, therefore, we investigated B cells at this site (29). Similar to the peritoneum and mesenteric lymph nodes, omentum B cell frequency and total numbers were reduced in the absence of NR2F6. B1a, B1b and B2 populations were also investigated in the omentum, B1a numbers and frequency were relatively low, although no differences were observed in *Nr2f6*-deficient mice relative to controls. A higher frequency of B1b cells was observed in the *Nr2f6^-/-^* mice but B1b cell numbers were not significantly altered. Finally, B2 cells were the largest population of B cells in the omentum and accounted for most of the B cell loss in the omentum of *Nr2f6*-deficient mice as both B2 frequency and total number were significantly reduced (**Figure 3F**). Together, our data show that at the sites investigated, B1 and B2 cell numbers in *Nr2f6*-deficient mice are unaffected in the spleen and inguinal lymph nodes, but B2 cells were reduced in the mesenteric lymph nodes and omentum, while B1b and B2 numbers were reduced in the peritoneum.

### *Nr2f6*-deficient bone marrow effectively repopulates B1b and B2 subsets in a competitive setting

**Figure 4:**
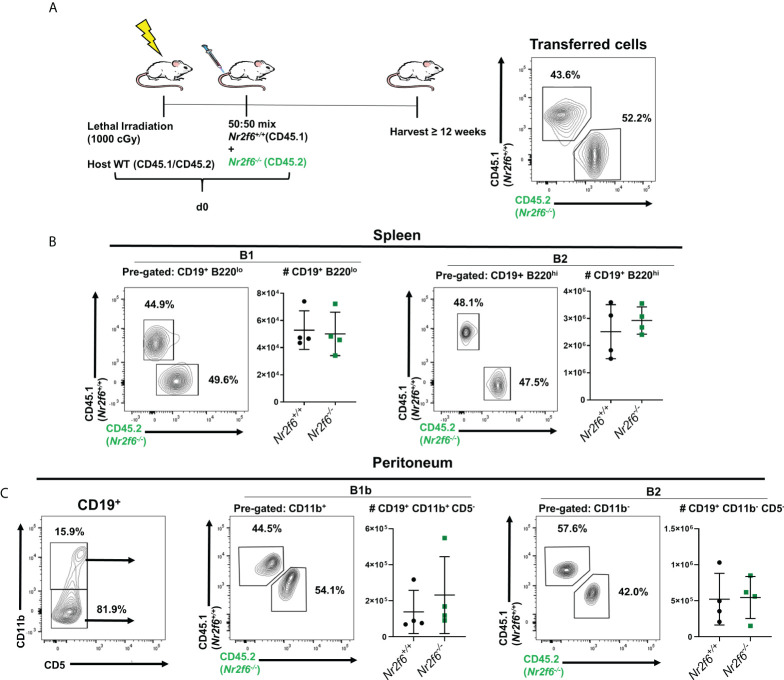
*Nr2f6*-deficient bone marrow cells reconstitute the B cell peritoneum population of wild-type hosts with the same efficiency as wild-type bone marrow. **(A)** Experimental scheme, CD45.1/CD45.2 host mice were lethally irradiated and on the same day a 50:50 mix of *Nr2f6^+/+^* (CD45.1) and *Nr2f6^-/-^* (CD45.2) bone marrow was transferred intravenously, reconstitution was allowed to occur for at least 12 weeks after which splenic and peritoneal B cell populations were identified by flow cytometry. (Far right) a representative CD45.1/CD45.2 stain of the bone marrow mix used for reconstitution. **(B)** After reconstitution, splenic B1 and B2 cells were investigated using CD19 and B220 expression. FACS plots show representative frequencies of each genotype and graphs show total cell counts of each population. **(C** left panel**)** Staining of CD11b and CD5 on CD19^+^ B cells isolated from the peritoneum. **(C** center and right panels**)** frequency and total numbers of *Nr2f6^+/+^* or *Nr2f6^-/-^* cells from the indicated peritoneal B cell population. Each data point represents an individual mouse, data shown are from three individual experiments. For most data sets statistical significance was determined using a two-tailed student t-test however, all data were tested for normality by Shapiro-Wilk testing, and for non-normally distributed data significance was determined using a Mann-Whitney U test. Within this figure, **(C)** B1b total cell count was not normally distributed and was tested by a Mann-Whitney U test, the data was not significantly different between the genotypes.

Because there were significant reductions in total *Nr2f6*-deficient B cells in some tissues but not others, we wanted to test if *Nr2f6*-deficient B cells at these sites would be at a disadvantage in a competitive environment relative to controls. Therefore, we performed a bone marrow chimera experiment, 12-week old CD45.1/CD45.2 heterozygous *Nr2f6^+/+^* recipient mice were lethally irradiated and a 50:50 mix of *Nr2f6^-/-^* (CD45.2^+^) and *Nr2f6^+/+^* (CD45.1^+^) bone marrow from age and gender-matched donors was transferred to the irradiated host mice (**Figure 4A**). Twelve weeks after transfer, B1 and B2 populations were investigated in the spleen and peritoneum by flow cytometry. Equivalent fractions of *Nr2f6^+/+^* and *Nr2f6^-/-^* B1 cells were found in the spleen and total counts were similar. *Nr2f6^+/+^* and *Nr2f6^-/-^* B2 cells were also found at equal numbers and frequencies in the spleens (**Figure 4B**). Previous reports have shown that adult bone marrow is capable of reconstituting both the B1b and the B2 compartments of the peritoneum, but not B1a, we also observed only B1b and B2 generation from adult bone marrow of both genotypes (6, 45) (**Figure 4C**, left panel). After reconstitution, the frequency of CD45.2^+^ and CD45.1^+^ cells within the peritoneal B1b and B2 populations was similar to the frequency at transfer (**Figures 4A, B**). Counts of both B1b and B2 were similar on average regardless of NR2F6 status of the cells (**Figure 4B**). These results suggest that within a competitive environment, *Nr2f6*-deficient B cells efficiently repopulate the spleen. Additionally, B cell-intrinsic loss of NR2F6 had no effect on B1b or B2 populations in the peritoneum of *Nr2f6^+/+^* mice and suggests that the development of these cells is not impaired by intrinsic *Nr2f6*-loss.

### *Nr2f6*-deficient B cells exhibit small reductions in CXCR5 and Beta7-integrin surface expression

One possible explanation for the reduced numbers of B2 and B1b cells in the *Nr2f6*-deficient peritoneal cavity may be altered migration to or retention in the peritoneum. Ansel et al. characterized the importance of CXCL13 and its receptor CXCR5 for both migration and retention of B cells in the peritoneum (28). Berberich et al. have demonstrated that the peritoneal milieu can induce upregulation of markers such as CXCR5, CXCR4 and Beta7-integrin on naïve splenic B cells transferred directly into the peritoneal cavity. They further demonstrate that these cells are more likely to rehome to the peritoneum after re-isolation and intravenous transfer to new hosts (30). We, therefore, investigated the possibility that reduced B cell numbers in the peritoneum of *Nr2f6^-/-^* mice is due to changes in the expression of these migration receptors.

We first harvested total peritoneum cells from *Nr2f6^+/+^* and *Nr2f6^-/-^* mice and determined CXCR4 expression on total B1 or B2 populations *via* flow cytometry. The expression pattern of CXCR4 was comparable on B1 and B2 cells isolated from both *Nr2f6*-deficient and -sufficient mice and the MFI of CXCR4 was also not altered between the genotypes (**Supplementary Figures 3A, B**). Similarly, no changes in the frequency of CXCR4^+^ B1 or B2 cells were found on *Nr2f6*-deficient cells relative to controls (**Supplementary Figure 3C**).

**Figure 5:**
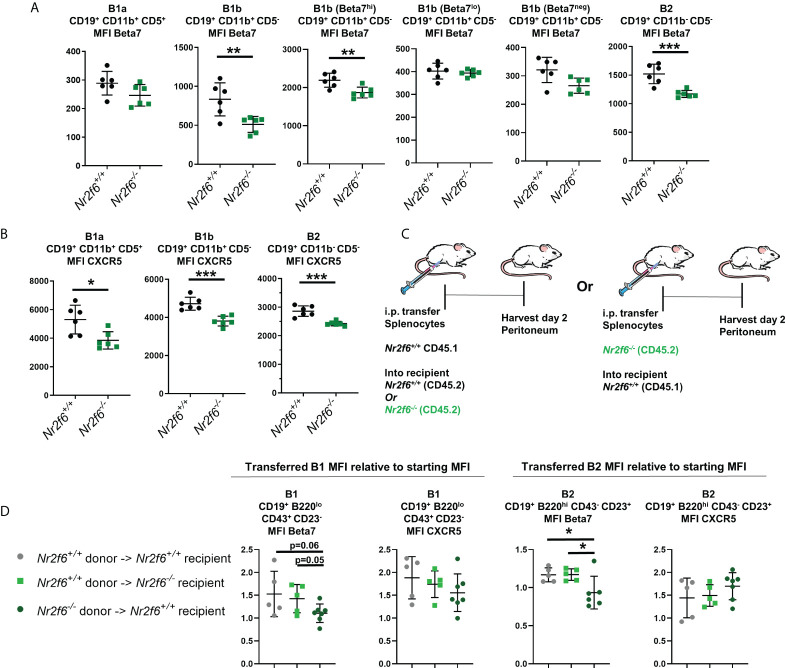
*Nr2f6^-/-^* peritoneal B cells have small but significant reductions in surface CXCR5 and Beta7 integrin, transferred *Nr2f6^+/+^* B cells do not exhibit the same expression pattern. **(A)** Flow cytometry was used to determine the mean fluorescence intensity (MFI) of Beta7-integrin on peritoneal B1a, B1b and B2 cells isolated from mice of the indicated genotype. B1b cells were investigated as a total population and separated into Beta7^hi^, Beta7^lo^ and Beta7^neg^ due to heterogeneous Beta7 expression on these cells (**Supplementary Figure 3**). **(B)** The MFI of CXCR5 was determined on B cell subsets isolated from mice of the indicated genotype. **(C)** Experimental scheme, CD45.1^+^ splenocytes were transferred into the peritoneum of either CD45.2^+^ *Nr2f6^+/+^* or CD45.2^+^ *Nr2f6^-/-^* recipients. Alternatively, CD45.2+ *Nr2f6^-/-^* splenic cells were transferred to the peritoneum of CD45.1+ *Nr2f6^+/+^* hosts. Cells were harvested two days later for analysis by flow cytometry. **(D)** The MFI of Beta7 and CXCR5 after harvest from the peritoneum, values displayed are normalized to the MFI of these markers determined prior to adoptive cell transfer. Each data point represents an individual mouse, data shown are from at least two individual experiments with two or more mice of each genotype per experiment. Statistical significance was determined using a two-tailed student t-test, significant p-values are displayed as *<0.05, **<0.01 and ***<0.001.

We next investigated the surface protein levels of Beta7-integrin on peritoneal B cells isolated from *Nr2f6*-sufficient or –deficient mice. Of note, B1a and B2 cells were uniform for Beta7 integrin staining, with B2 cells expressing elevated Beta7-integrin and B1a cells staining only weakly. B1b cells, on the other hand, were heterogeneous with three distinct populations, which we defined as Beta7^hi^, Beta7^lo^ and Beta7^neg^ (**Supplementary Figure 4B**). Thus, the MFI of Beta7-integrin was determined on total B1a and B2 populations, while B1b cells were divided into total B1b, Beta7^hi^, Beta7^lo^ or Beta7^neg^ for analysis of MFI. B1a Beta7 MFI was not significantly altered between the genotypes. In contrast, Beta7 MFI on both total *Nr2f6^-/-^* B1b, as well as the B1b Beta7^hi^ populations, was reduced, while the MFI of Beta7 on the Beta7^lo^ and Beta7^neg^ cells was not significantly altered on *Nr2f6*-deficient cells (**Figure 5A**, second and third panels from the right). We found that the Beta7 MFI on B2 cells obtained from *Nr2f6^-/-^* mice was also significantly reduced (**Figure 5A**, far right panel). However, average reductions in Beta7 MFI on B1b and total B2 cells were relatively minor, with the *Nr2f6*-deficient B1b Beta7^hi^ population showing an average reduction of only 14.7%. While the average loss of Beta7 on total *Nr2f6*-deficient B1b cells was larger, but still relatively modest, with a 38% reduction in the MFI. B2 cells also showed a relatively minor Beta7 reduction on *Nr2f6^-/-^* cells with an average MFI decrease of 23%. Similarly, CXCR5 expression was significantly reduced on *Nr2f6^-/-^* B1b and B2 cells, but also B1a cells (**Figure 5B**). Although, like Beta7 staining, reduction of CXCR5 expression was relatively minor with B2 cells isolated from *Nr2f6*-deficient mice exhibiting on average ~15% lower CXCR5. B1a displayed the highest CXCR5 difference with on average 27% lower expression on cells from *Nr2f6^-/-^* mice.

We also investigated the frequency of Beta7^+^ B1 and B2 cells and found that the overall Beta7 staining pattern on B cells was mirrored in the quantification of the frequencies of each population. With no significant changes to the percentage of Beta7^+^ B1a cells but a significant reduction in Beta7^+^ *Nr2f6^-/-^* B2 cells (**Supplementary Figures 4A, 4C**). Similarly, the total population of *Nr2f6^-/-^* B1b cells was shifted toward the Beta7^neg^ gate with a lower fraction of Beta7^hi^ but more Beta7^neg^ cells (**Supplementary Figure 4B**). A similar small but significant reduction of surface CXCR5 was seen when the frequency of CXCR5^+^ cells was determined on *Nr2f6^-/-^* B1a, B1b and B2 peritoneal B cells (**Supplementary Figure 5A–C**).

As previously mentioned, the peritoneal milieu has been shown to upregulate the expression of CXCR5 and Beta7 on splenic B cells transferred to the peritoneum (30). In order to investigate possible defects of the *Nr2f6*-deficient peritoneal milieu in driving expression of these markers; we performed adoptive transfer of CD45.1^+^ *Nr2f6^+/+^* splenocytes into the peritoneum of CD45.2^+^ *Nr2f6^+/+^* or CD45.2^+^ *Nr2f6^-/-^* recipients (**Figure 5C**). Total peritoneal cells were isolated from recipient mice two days after transfer and surface expression of CXCR5 and Beta7 were determined by flow cytometry, the MFI determined on day two after transfer was then normalized to the MFI of the donor cells measured prior to adoptive cell transfer. Total transferred B1 cells were defined as B220^lo^, CD19^+^, CD43^+^ and CD23^-^, these cells both prior and post-transfer consisted of ~90% B1a as measured by CD5 expression (**Supplementary Figure 6**). Due to the small B1 population the B1 cells were not divided into B1a and B1b as too few cells were present to provide accurate results. We found that transferred *Nr2f6^+/+^* B1 and B2 cells in both *Nr2f6^+/+^* and *Nr2f6^-/-^* recipients had similar induction of CXCR5 and Beta7 (**Figure 5D**) indicating that under these conditions, the *Nr2f6*-deficient peritoneal milieu was able to promote efficient expression of CXCR5 and Beta7. To test for a B cell-intrinsic role of *Nr2f6* in the expression CXCR5 and Beta7, total CD45.2^+^ *Nr2f6^-/-^* splenocytes were transferred to the peritoneum of CD45.1^+^ *Nr2f6^+/+^* recipient mice (**Figure 5C**). Again, peritoneum cells were isolated from recipient mice two days post transfer and the MFI normalized to donor cell levels measured before transfer. Upregulation of CXCR5 appeared unaffected by B cell intrinsic NR2F6-loss when compared to CD45.1^+^ *Nr2f6^+/+^* transferred to either genotype of recipient. However, transferred *Nr2f6^-/-^* B1 cells generally failed to induce Beta7, while *Nr2f6^-/-^* B2 cells both failed to induce Beta7 but slightly reduced expression of this migration marker (**Figure 5D**).

Our data demonstrate a small but significant reduction of CXCR5 and Beta7 on peritoneal B cells isolated from *Nr2f6*-deficient mice. Our transfer experiments suggest a B cell-intrinsic role of NR2F6-loss for the reduction of Beta7 rather than changes in the peritoneal milieu of *Nr2f6*-deficient animals. While, changes to CXCR5 induction were not observed in any condition. The data suggest that the factors causing lower CXCR5 expression by *Nr2f6*-deficient peritoneal B cells are complex and defects may be due to role for *Nr2f6* in long-term expression rather than short-term induction.

### Peritoneal *Nr2f6* ^-/-^ B cells show similar proliferation but enhanced apoptosis relative to controls

**Figure 6:**
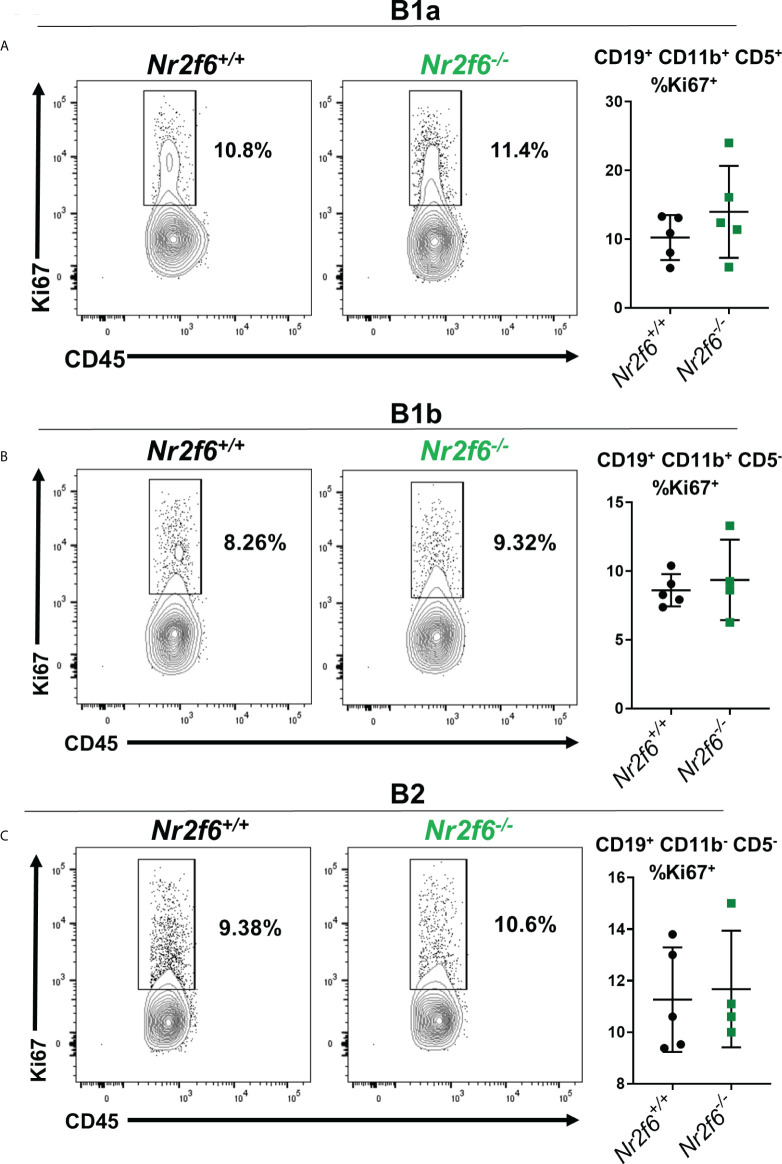
*Nr2f6^-/-^* peritoneal B cells exhibit similar Ki67 staining as wild-type cells. Intracellular Ki67 staining was performed *ex vivo* on **(A)** B1a, **(B)** B1b and **(C)** B2 cells, isolated from mice of the indicated genotype B cell populations were defined using CD19, CD11b and CD5. The left panels display representative flow cytometry plots and graphs (right side) show the percentage of each population staining positively for Ki67 from all experiments. Data shown are derived from two individual experiments with at least two mice. Each data point represents an individual mouse. Statistical significance was determined using a two-tailed student t-test, data were not significantly different between the genotypes.

Because migration markers were not drastically altered on *Nr2f6^-/-^* peritoneal B cells, we investigated other potential causes of the observed phenotype. A lower proliferation rate could explain the reduced peritoneal B cells in *Nr2f6*-deficient mice. Therefore, proliferation was investigated on peritoneal B1a, B1b and B2 cells by intracellular staining for Ki67. We found that B1a, B1b and B2 cell populations from wild-type and *Nr2f6^-/-^* mice displayed similar fractions of Ki67^+^ cells, suggesting similar proliferation rates between the genotypes (**Figures 6A–C**).

**Figure 7:**
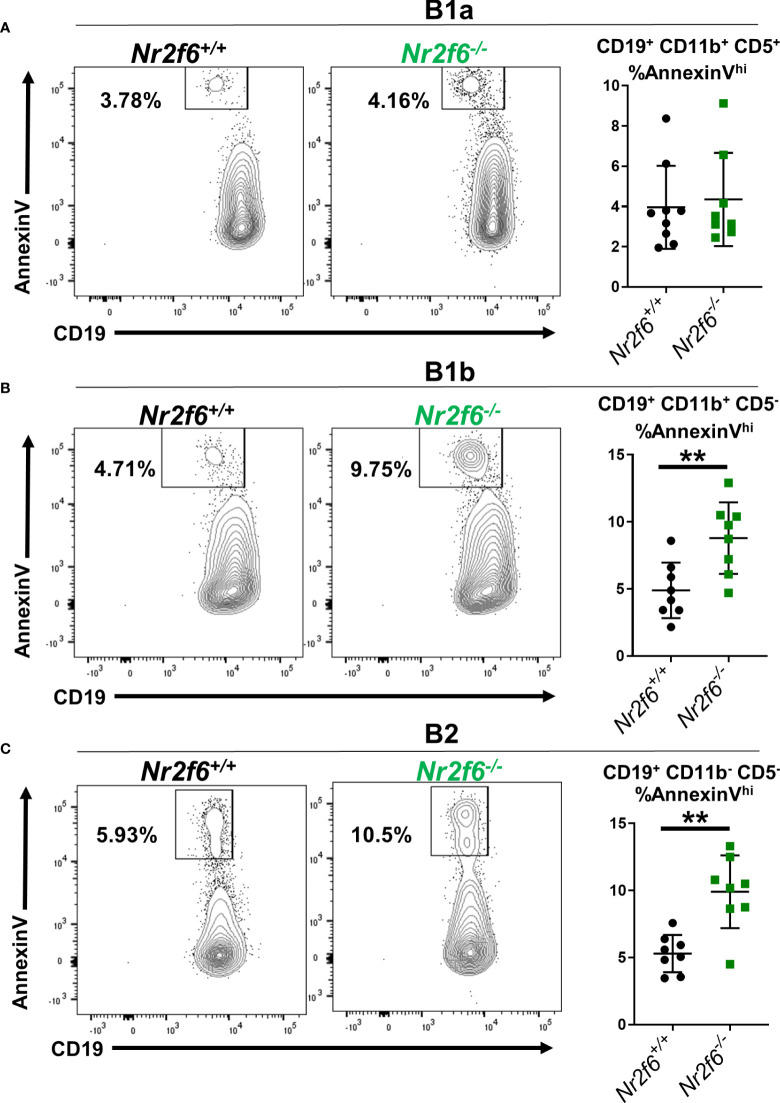
The frequency of *Nr2f6^-/-^* peritoneal B1b and B2 cells staining for AnnexinV is higher relative to *Nr2f6^+/+^* peritoneal B cells. Peritoneal cells were isolated and B cell populations defined by CD5, CD11b and CD19 expression. The frequency of AnnexinV staining within **(A)** B1a, **(B)** B1b and **(C)** B2 cells is displayed. FACS panels show representative FACS plots and graphs (right side) show the frequency of AnnexinV staining from all experiments performed. Each data point represents one mouse. The experiment was performed three times with at least two mice of each genotype per experiment. Statistical significance was determined using a two-tailed student t-test, significant p-values are displayed as **<0.01.

An alternative is that *Nr2f6*-deficient peritoneal B1b and B2 cells are more susceptible to apoptosis. We investigated apoptosis of the B cell subsets using AnnexinV staining. B1a cells from both *Nr2f6^+/+^* and *Nr2f6^-/-^* mice showed a relatively low fraction of apoptotic AnnexinV^hi^ cells. But, no significant difference was observed in AnnexinV staining of *Nr2f6^-/-^* B1a cells compared with controls (**Figure 7A**). In contrast, *Nr2f6^-/-^* B1b and B2 cells displayed a significantly higher fraction of AnnexinV^hi^ cells, thus *Nr2f6*-deficient B1b and B2 cells were significantly more apoptotic than control cells (**Figures 7B, C**). We confirmed that our CD19^lo^ AnnexinV^+^ population was not inadvertently including non-B cell populations such as monocytes by comparing CD19 by AnnexinV staining on all ungated events and found no overlap of the CD19^lo^ AnnexinV^hi^ population with any other major population (**Supplementary Figure 7A**, left panel). Addtionally, we validated our AnnexinV^hi^ gate by co-labeling AnnexinV stained cells with the viability dye DAPI; we find that only the highest AnnexinV staining cells co-label with DAPI (**Supplementary Figure 7A**, right panel). In **Figure 2**, we found lower frequency and total cell numbers of CD11b^-^ CD19^+^ B220^lo^ cells in the PerC, we therefore, investigated whether survival was reduced in these cells by AnnexinV staining. Like the B1b and B2 cells, we again found an increased frequency of AnnexinV^+^ CD11b^-^ CD19^+^ B220^lo^ cells isolated from *Nr2f6*-deficient mice relative to controls (**Supplementary Figure 7B**). A similar increase in AnnexinV staining was observed on CD5^+^ CD11b^-^ CD19^+^ B220^lo^ cells from *Nr2f6^-/-^* mice, suggesting that reduced survival may play a role in the loss of these cells as well (**Supplementary Figure 7C**).

**Figure 8:**
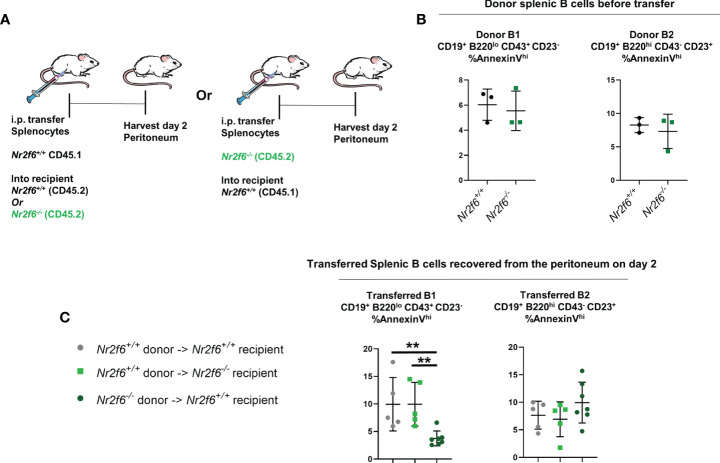
Transfer of *Nr2f6*-sufficient or -deficient splenocytes into both *Nr2f6^+/+^* and *Nr2f6^-/-^* recipients. **(A)** Experimental scheme for adoptive cell transfer. **(B)** Frequency of AnnexinV^+^ cells from the indicated donor splenocytes measured prior to adoptive cell transfer. **(C)** AnnexinV^+^ frequency on transferred B1 and B2 cells, two days after transfer. Data shown are from three independent experiments with two or more mice of each genotype per experiment. Statistical significance was determined using a two-tailed student t-test, significant p-values are displayed as **<0.01.

Finally, we investigated AnnexinV staining on transferred splenic B cells in the peritoneum using the same adoptive transfer of total splenocytes and harvest scheme as in **Figure 4C** (**Figure 8A**). Donor *Nr2f6^+/+^* and *Nr2f6^-/-^* splenocytes were stained for AnnexinV prior to transfer and no differences were observed on the B cell populations (**Figure 8B**). On day two, transferred splenocytes were recovered from the peritoneum of recipient mice and the B1 and B2 cells investigated. Again, due to the low number of transferred splenic B1 cells we did not divide this population into the individual B1a or B1b subsets. Interestingly, transferred *Nr2f6*-deficient splenic B1 cells were less apoptotic than their wild-type counterparts, regardless of the genotype of recipient. But, no differences in AnnexinV staining was observed between *Nr2f6^+/+^* or *Nr2f6^-/-^* transferred B2 cells (**Figure 8C**). Altogether, these data suggest that proliferation is unaltered in B cells residing in the peritoneum of *Nr2f6*-deficient mice, but that survival of long-term resident B1b and B2 cells and precursor CD11b^-^ B1 cells is impaired. While the adoptive transfer experiments did not reveal a specific role for either the *Nr2f6^-/-^* milieu or a cell-intrinsic role for *Nr2f6* in increased cell death. Together, the data suggests that *Nr2f6*-loss may not acutely affect the survival of B1b or B2 cells, but in the context of resident cells, NR2F6 may positively affect their lifespan.

## Discussion

Peritoneal B cells offer a first line of defense against pathogens originating from the intestinal tract. B2 cells have broad reactivity towards pathogens, while B1 cells are enriched for lipid binding and are critical for clearance of specific pathogens such as *Streptococcus pneumonia* and *Enteropathogenic E. coli* (EPEC), among others (1, 46). Both B1 and B2 cells can contribute to long-term protection from pathogens (3, 4). We demonstrate with this study a role for the nuclear receptor NR2F6 in the homeostasis of B1b and B2 cells within the peritoneal cavity, while B1a cells are not affected by NR2F6-loss. B1 and B2 cell numbers were unaltered in some immune organs of *Nr2f6*-deficient mice, including spleen and inguinal lymph nodes, while significant reductions were observed in the peritoneum and associated sites such as mesenteric lymph nodes and omentum, suggesting a possible site-dependent effect of NR2F6-loss.

A previous report from our group identified NR2F6 as a critical positive regulator of *Muc2* production. Loss of NR2F6 resulted in reduced intestinal barrier function, increased susceptibility to dextran sodium sulfate (DSS) induced colitis and spontaneous late-onset colitis (47). Changes in inflammation or bacterial component presence within the *Nr2f6*-deficient peritoneum could explain the observed reduction in B cell numbers, either *via* increased activation and migration or activation-induced cell death. Although, the data generated in this report indicate this is an unlikely scenario, given that numbers of innate cells were not increased (or decreased) in the peritoneum of *Nr2f6^-/-^* mice. For example, neutrophils are rapidly recruited to the peritoneum after lipopolysaccharide (LPS) injection and were unchanged in *Nr2f6*-deficient mice (48). Additionally, Ghosn et al. have defined two main macrophage populations within the peritoneum a minor population termed small peritoneal macrophages and a predominate population termed large peritoneal macrophages. Large peritoneal macrophages are sensitive to inflammation and are rapidly lost after LPS or thioglycolate stimulation, while small peritoneal macrophages accumulate in this context (43). In the *Nr2f6*-deficient peritoneum, total large peritoneal macrophages and small peritoneal macrophages as determined by F4/80 and MHCII expression, were not significantly altered. Ghosn and colleagues additionally identify monocytes infiltrating in response to LPS injection as the precursors to small macrophages. However, we were unable to detect any significant changes in either small macrophage or peritoneal monocyte numbers from *Nr2f6^-/-^* mice relative to *Nr2f6^+/+^* controls. In addition, a previous study has shown that activation of CD5^+^ B1 cells *via* TLR ligands results in reduced CD5 expression, we observed elevated fractions of CD5^+^ cells in the peritoneum of *Nr2f6*-deficient mice, indicating that significant levels of TLR ligands are not available to CD5 expressing B cells (49). Together our data indicates that substantial inflammation is likely not present in the peritoneum of young *Nr2f6*-deficient mice.

We found with this study that survival of B1b and B2 cells in the *Nr2f6^-/-^* peritoneum as measured by AnnexinV was significantly reduced, but the survival of B1a cells was unaltered. Previous studies by our group have extensively linked NR2F6 to the control of secreted factors, particularly cytokines, in the context of T cell activation (38, 39). In general the factors that promote B1 and B2 cell survival in the peritoneum are not well charactherized, however, several secreted factors have been described to control the survival of peritoneal B cells, although none have yet been linked to NR2F6. BAFF can reduce FcγIIRb levels, decreasing death mediated by this receptor (21). While one study has suggested that secreted natural IgM can affect BCR signaling and lead to the death of peritoneal B cells, presumably due to excessive activation. Interestingly, BAFF cannot rescue death induced by natural IgM on peritoneal B cells (26). Survival likely contributes to the reduced numbers of B1b and B2 cells in the *Nr2f6*-deficient PerC, but exactly how *Nr2f6*-loss contributes to reduced survival is not clear.

In accordance with previous reports, we find that adult bone marrow was unable to generate significant B1a cell numbers (6, 10). However, *Nr2f6*-deficient bone marrow was able to reconstitute the B1b and B2 peritoneal populations of lethally irradiated wild-type mice at the same rate as *Nr2f6*-sufficient bone marrow. In line with this observation, our group has previously reported no block in B cell development of NR2F6-null mice (34). Our data presented here indicates that NR2F6-loss does not have a specific impact on the development of peritoneal B1b or B2 cells.

Work by the Cyster group demonstrated a critical role for the CXCR5 ligand CXCL13 in the peritoneal homing of B1 and B2 cells (28). While work by Berberich et al. has shown that direct transfer of splenic B cells into the peritoneum is sufficient to induce CXCR5 and Beta7-integrin upregulation on naïve splenic B cells and increased peritoneal homing of these cells after i.v. transfer to new mice, suggesting a site-specific signal or signals are required for peritoneal B cells to fully express migration molecules required for peritoneum homing/retention (30). We found that B cells isolated from the peritoneum of *Nr2f6*-null mice had significantly reduced CXCR5 and Beta7-integrin surface expression. Although, these reductions were relatively minor, with *Nr2f6^-/-^* B cell populations expressing at most an average reduction of 38% less surface Beta7 while CXCR5 reductions were on average even smaller. The previously mentioned study by the Cyster group observed normal numbers of peritoneal B cells in CXCL13 heterozygous mice, suggesting that the minor reduction of CXCR5 on *Nr2f6*-deficient B cells is likely not solely responsible for lower B cell numbers in the peritoneum (28). In addition, the largest reduction of CXCR5 on *Nr2f6*-deficient peritoneal B cells was found on B1a cells despite normal numbers of these cells, arguing against a major role for this marker in reduced B1b and B2 cell numbers at this site. However, these experiments still do not completely rule out a minor role for Beta7 or CXCR5 in reduced peritoneal B1b and B2 cells in the absence of *Nr2f6*.

Whether the observed reduction of B1b and B2 cells in the peritoneum of *Nr2f6*-null mice is primarily due to *Nr2f6*-intrinsic or -extrinsic loss is less clear. We found that generation of B1b and B2 cells from *Nr2f6*-deficient bone marrow is as efficient as from wild-type bone marrow, and numbers of *Nr2f6^+/+^ and Nr2f6^-/-^* B1b and B2 cells are similar in wild-type hosts, which would suggest a primarily extrinsic role for *Nr2f6* in reduced peritoneal B1b and B2 cells. However, adoptive cell transfer experiments, in which *Nr2f6*-sufficient splenocytes were transferred into the peritoneum of *Nr2f6*-deficient recipients, showed no effect on either B cell survival or expression of CXCR5 or Beta7. These results may indicate that the effect of reduced survival of *Nr2f6^-/-^* B1b and B2 cells is not an acute phenomenon as these cells were harvested only 48 hours after transfer but could suggest a role for long-term survival within the peritoneum. Because *Nr2f6^-/-^* splenic B cells transferred to the *Nr2f6^+/+^* PerC failed to induce Beta7, it may be that *Nr2f6* acts in both a B cell intrinsic and extrinsic manner resulting in a combination of reduced retention and increased cell death in the peritoneum, that ultimately reduces B1b and B2 cells numbers.

Interestingly, the TRANSFAC^®^ database predicts that both CXCR5 and Beta7 have NR2F6 binding sites within their respective promotors, which supports a role for NR2F6 in direct regulation of these markers and our adoptive transfer data supports this conclusion for Beta7. Additionally, based on the TRANSFAC^®^ database no putative NR2F6 binding site is present in the CXCR4 promotor, in line with our observation that NR2F6-loss had no effect on this receptors expression. Given previous reports of undetectable transcript for *Nr2f6* in the peritoneal B1 and B2 populations the relatively mild reduction in both CXCR5 and Beta7-integrin in the absence of NR2F6 is perhaps not surprising, it may be that a role for NR2F6 is limited to specific situations (50).

The data presented here, identifies a complex role for NR2F6 in the control of peritoneal B cell homeostasis, particularly survival and control of CXCR5 and Beta7 expression by the B1b and B2 subsets. Further work is warranted to define the specific B cell-extrinsic and -intrinsic roles of *Nr2f6* in B1b and B2 cell homeostasis, such as how intrinsic *Nr2f6* regulates migration markers such as Beta7 and to what extent this contributes to the phenotype. Additionally, why B cells in the spleen are found at equivalent numbers in *Nr2f6^-/-^* mice relative to *Nr2f6^+/+^* mice indicates some specific factors controlled by NR2F6 are critical for peritoneal B1b and B2 cells but not B cells in other locations. Similarly, defining the factors that affect B1b and B2 cell homeostasis in the peritoneum but not the B1a subset will be of interest.

## Data availability statement

The original contributions presented in the study are included in the article/**Supplementary Material**. Further inquiries can be directed to the corresponding authors.

## Ethics statement

The animal study was reviewed and approved by the Austrian Bundesministerium Bildung Wissenschaft und Forschung.

## Author contributions

This study was designed by WO and NH-K, with input from ED. Experiments were performed by WO, with assistance from BJ, VL, JW and NH-K. Data analysis was performed by WO and NH-K. Experimental results were interpreted and discussed by WO, NH-K, GB and ED. The manuscript was written by WO with input from NH-K. All authors contributed to editing of the manuscript and have approved the final version.

## Funding

This work was supported by the FWF (Austrian Science Fund) with the following grant (P28694-B30; DOC82) and the Tirolean Science Fund (F.30904/7-2021) awarded to NK. GB was supported by a European Research Council (ERC) grant and an FWF grant (P31383-B30). ED was supported by an early-stage funding grant provided by the University of Innsbruck (2017/BIO-5), a Tirolean Science Fund grant (UNI-0404/2310) and an Action D Swarovski grant (2016/BIO-20). VL was also supported by an FWF grant (P32755).

## Acknowledgments

We are thankful for the technical assistance of Nadja Haas and Martin Heitz.

## Conflict of interest

The authors declare that the research was conducted in the absence of any commercial or financial relationships that could be construed as a potential conflict of interest.

## Publisher’s note

All claims expressed in this article are solely those of the authors and do not necessarily represent those of their affiliated organizations, or those of the publisher, the editors and the reviewers. Any product that may be evaluated in this article, or claim that may be made by its manufacturer, is not guaranteed or endorsed by the publisher.
