# Supplementary material for: A role for the nuclear receptor NR2F6 in peritoneal B cell homeostasis
Source: Front Immunol. 2022 Aug 16;13:845235. doi: 10.3389/fimmu.2022.845235 (PMC9425112; doi:10.3389/fimmu.2022.845235)
Supplement: Supplementary file 1 [file Presentation_1.pdf]

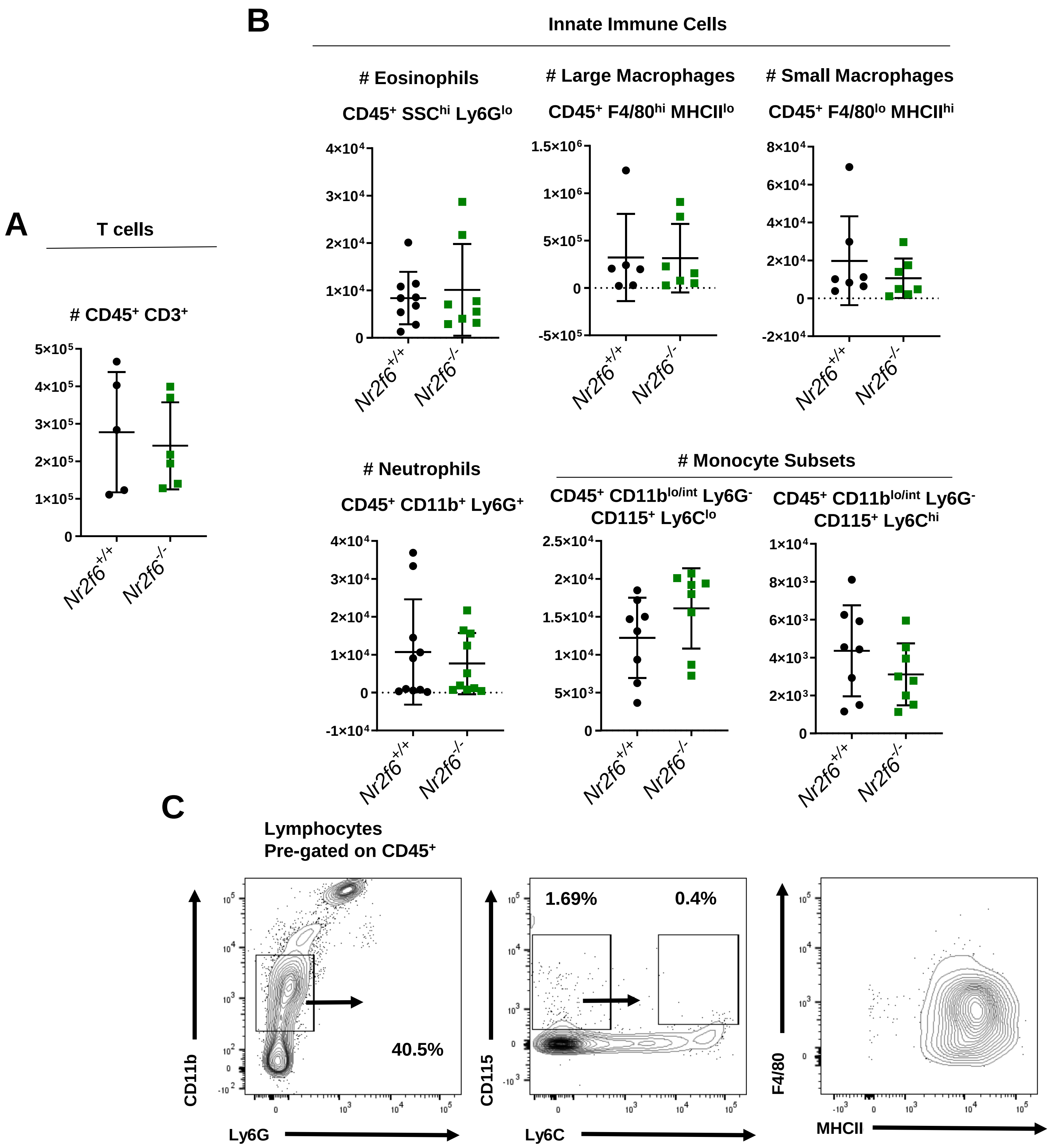

Supp Fig 1: T cell and Innate populations are similar in the PerC of *Nr2f6*-sufficient and -deficient mice.

(A) Frequency of T cells was determined by flow cytometry staining for CD45 and CD3 were used to calculate total T cell numbers. **(B)** Similarly, innate cells were defined based on CD45 staining in conjunction with the above indicated markers and total peritoneum cell numbers were used to calculate the total numbers of each population. **(C)** The gating strategy used to define the monocyte subsets is shown, the far right panel displays the F4/80 and MHCII staining pattern on pre-gated non-inflammatory monocytes, to show that small peritoneal macrophages were not inadvertently included in this gate. Each data point represents an individual mouse, data shown are from at least two individual experiments with at least two mice of each genotype per experiment. For most data sets statistical significance was determined using a two-tailed student t-test however, all data were tested for normality by Shapiro-Wilk testing, and for non-normally distributed data, significance was determined using a Mann-Whitney U test Within this figure, **(B)** Eosinophil counts and large peritoneal macrophage counts were not normally distributed and thus tested by a Mann-Whitney U test, no populations were significantly different between the genotypes.

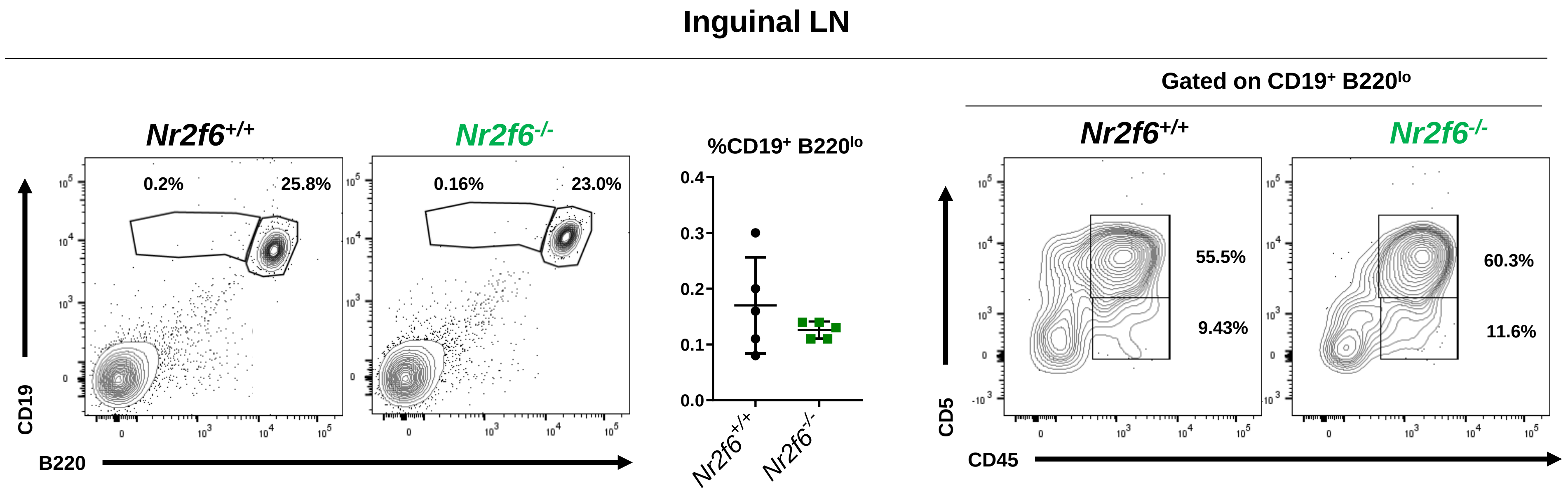

Supp Fig 2: Investigation of B1 cells in the inguinal lymph node of *Nr2f6*$^{-/-}$ and *Nr2f6*$^{+/+}$ mice.

Single inguinal lymph nodes were harvested and single cell suspensions stained for CD19 and B220. Total B1 cells were defined as CD19^{+} B220^{lo} the frequency of B1 cells from five individual mice is shown in the middle panel. Representative FACS panels defining B1 cells by CD43 and CD5 staining is shown in the right two panels. Statistical significance was investigated using a student t-test, B1 frequency was not significantly different between the genotypes.

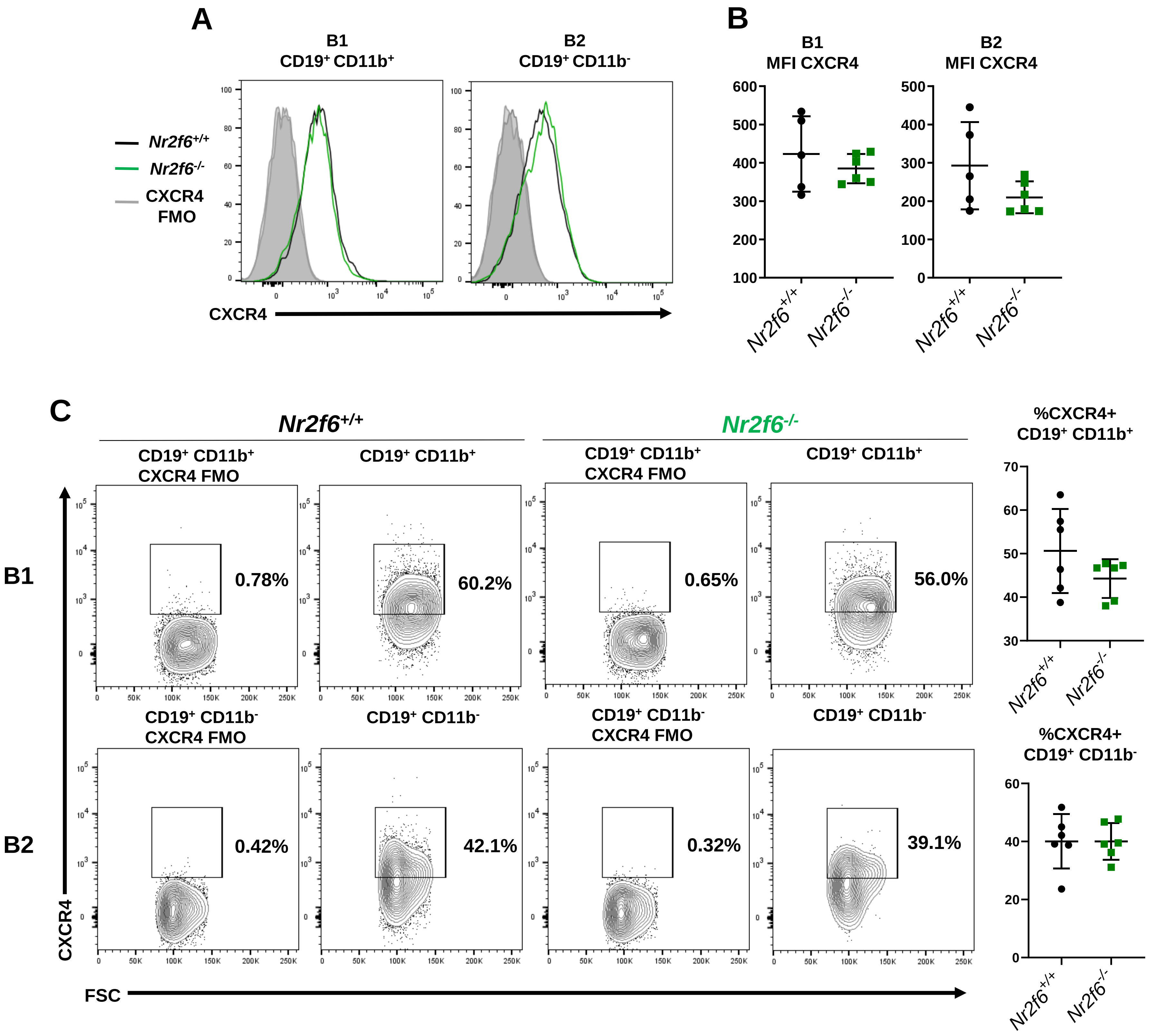

Supp Fig 3: CXCR4 is similarly expressed on peritoneal B cells from *Nr2f6*$^{+/+}$ and *Nr2f6*$^{-/-}$ mice.

Total peritoneal cells were isolated and CXCR4 expression was measured by flow cytometry. B cells were first determined by CD19 gating and B1 and B2 populations further divided by CD11b expression. **(A)** Histograms show CXCR4 intensity relative to FMO controls for total B1 and B2 cells. **(B)** The MFI of B1 and B2 cells from all mice investigated are plotted. **(C)** The frequency of CXCR4^{+} cells is shown, gates were determined using the FMO controls. Data shown is from at least two individual experiments with at least two mice of each genotype per experiment. Each data point represents an individual mouse, data shown are from at least two individual experiments with two or more mice of each genotype per experiment. Statistical significance was determined using a two-tailed student t-test, data were not significantly different between the genotypes.

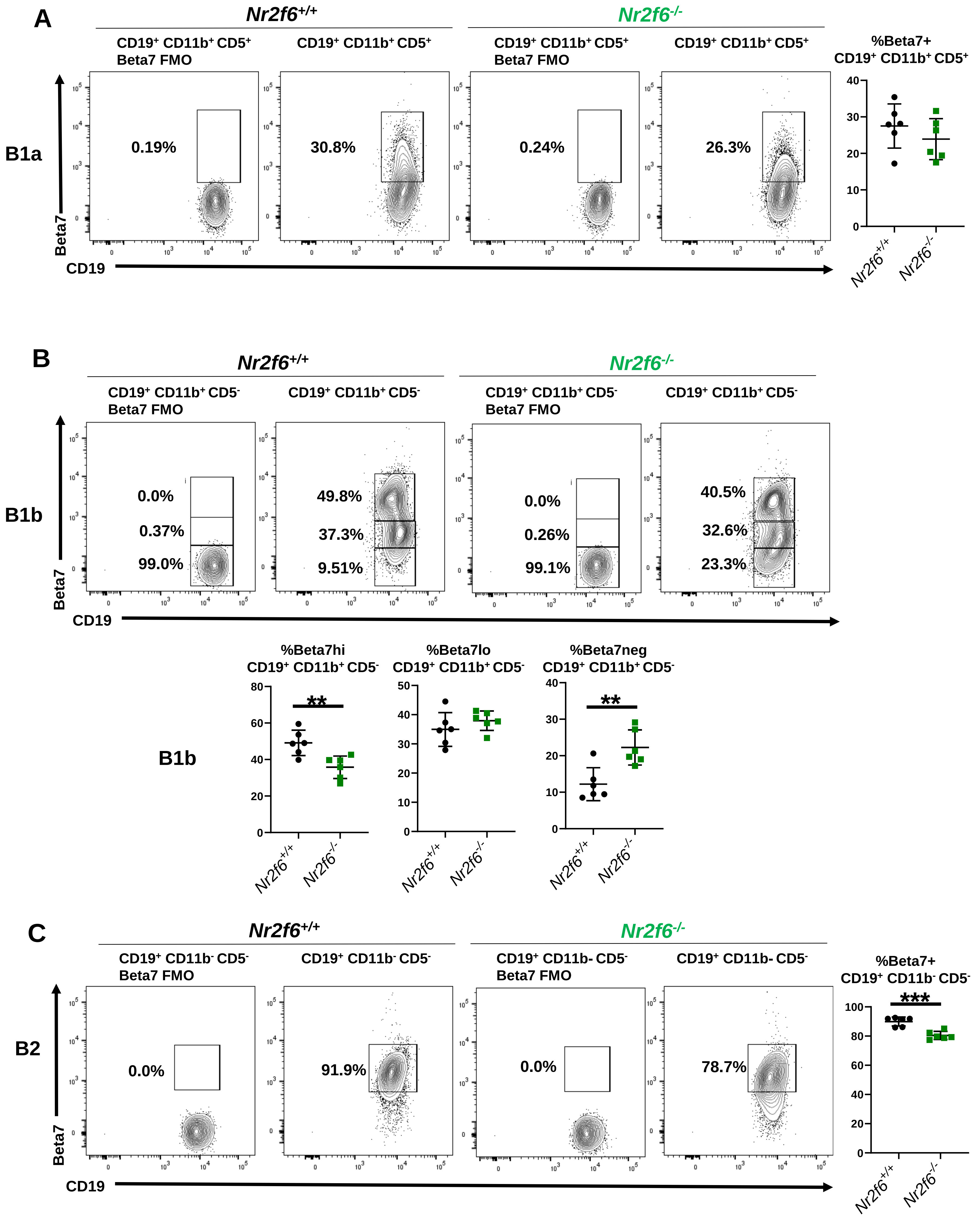

Supp Fig 4: Beta7-integrin staining patterns differ on B1a, B1b and B2 peritoneal B cells from *Nr2f6$^{-/-}$* and *Nr2f6$^{+/+}$* mice.

Representative FACS plots showing the Beta7-integrin staining patterns on B1a, B1b and B2. **(A)** The frequency of Beta7^{+} B1a cells was determined using the FMO controls, representative FACS plots are shown and data on the right summarizes the frequency of Beta7^{+} B1a cells in all experiments performed. **(B)** Three distinct populations with varying levels of Beta7-integrin were found in the total B1b population. Representative FACS plots are shown and the frequency of each population as determined in all experiments is represented in graphs below the FACS plots. **(C)** Representative FACS plots from the B2 population and the frequency Beta7^{+} B2 cells from all mice investigated are shown. Each data point represents an individual mouse, data shown are from at least two individual experiments with two or more mice of each genotype per experiment. Statistical significance was determined using a two-tailed student t-test, significant p-values are displayed as **<0.01 and ***<0.001.

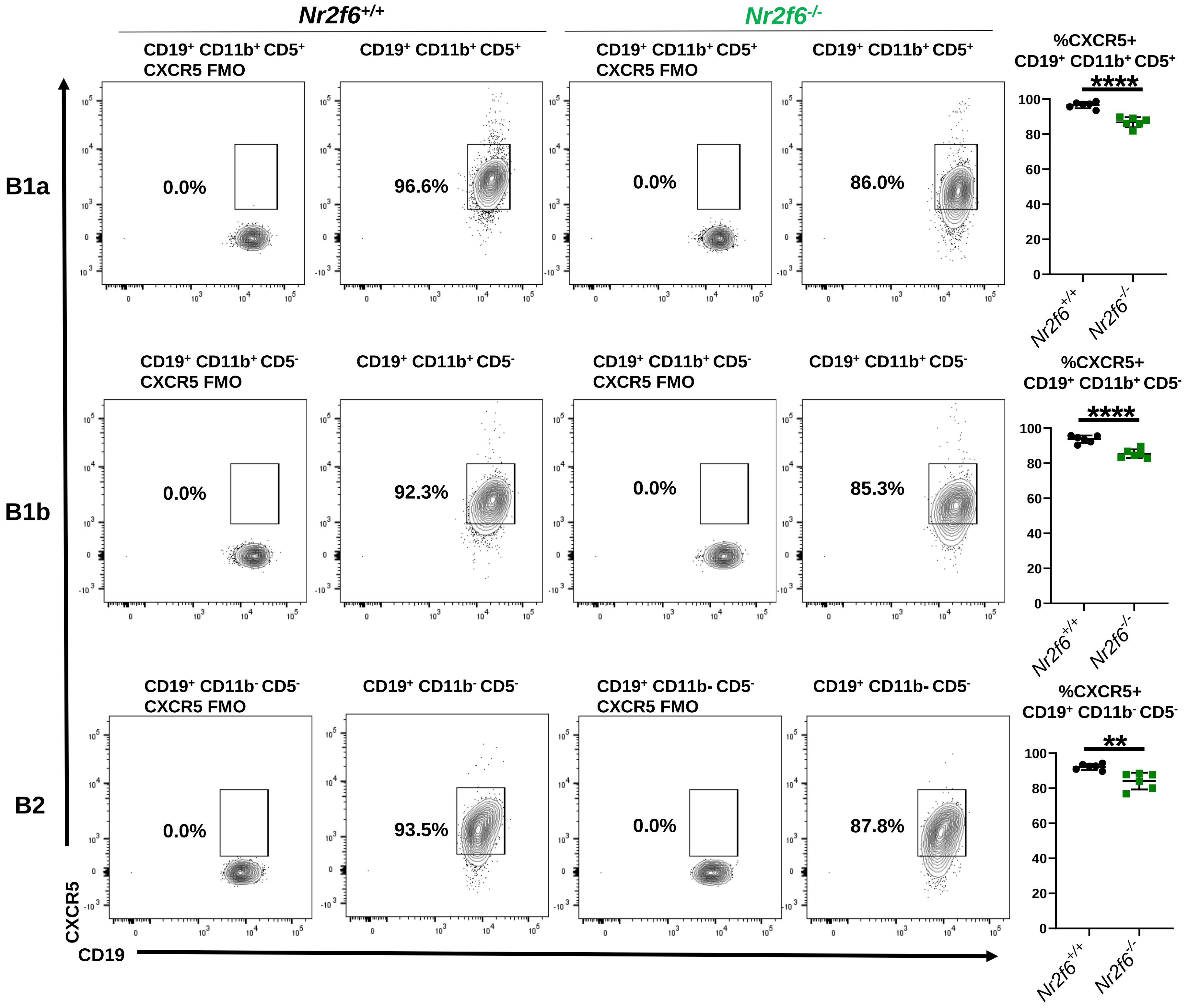

Supp Fig 5: Reduced CXCR5 frequency on all peritoneal B cells isolated from *Nr2f6*$^{-/-}$ mice.

Representative FACS plots showing CXCR5 staining and FMO controls on B1a, B1b and B2 cells isolated from the peritoneum of *Nr2f6*$^{+/+}$ or *Nr2f6*$^{-/-}$ mice. On the right side the frequency of CXCR5^{+} cells is shown for each cell type and for all mice investigated. Each data point represents an individual mouse, data shown are from at least two individual experiments with two or more mice of each genotype per experiment. Statistical significance was determined using a two-tailed student t-test, significant p-values are displayed as **<0.01 and ****<0.0001.

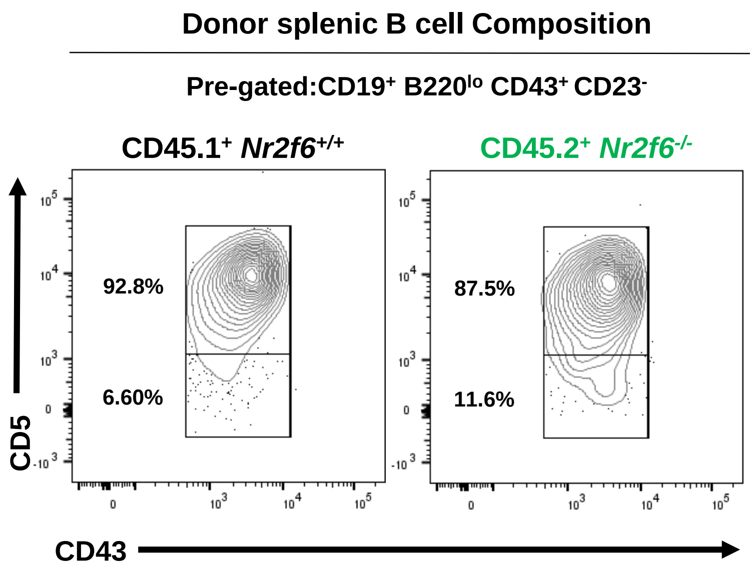

Supp Fig 6: Composition of the donor splenic B1 populations.

B1 cells from the spleens of indicated donor mice were initially defined as $CD19^{+}$ and $B220^{lo}$ and B1a or B1b cells determined using CD5 and CD43. Plots shown are representative of three individual animals of each genotype.

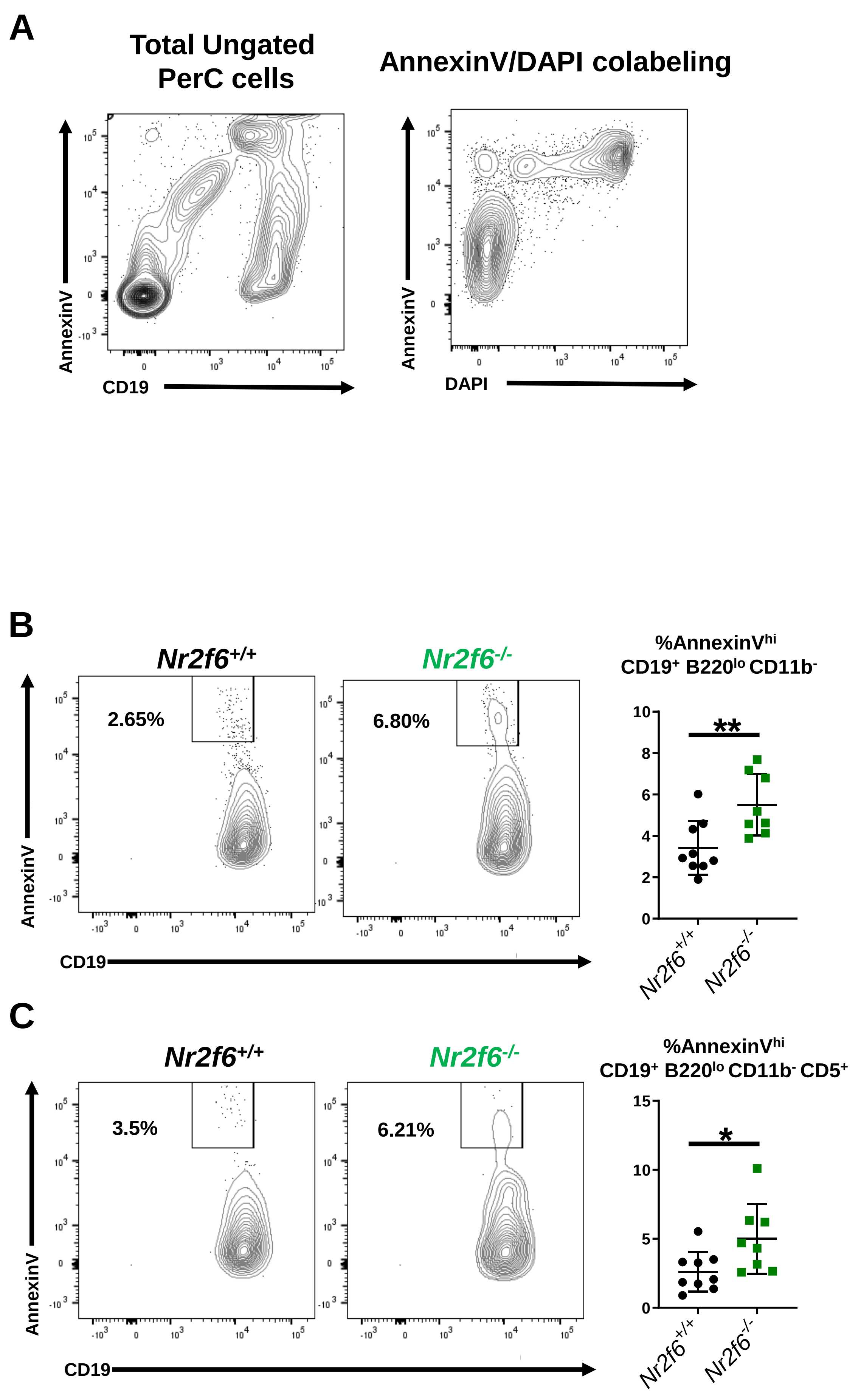

Supp Fig 7: Additional AnnexinV staining information and *Nr2f6*-deficient CD19$^+$ B220^{lo} CD11b$^-$ cells display increased AnnexinV staining.

(A) Total events displayed by AnnexinV and CD19 (left panel) and AnnexinV staining with the viablilty dye DAPI is shown on splenic B2 cells activated for four days *in vitro* with LPS. **(B)** Representative FACS plots showing AnnexinV staining on CD19$^+$ B220^{lo} CD11b$^-$ cells isolated from the peritoneum of *Nr2f6*$^{+/+}$ or *Nr2f6*$^{-/-}$ mice. On the right side the frequency of AnnexinV$^+$ cells is shown for each mouse investigated. **(C)** AnnexinV$^+$ frequency on CD19$^+$ B220^{lo} CD11b$^-$ CD5$^+$ cells are shown by representative FACS plots and a summary of all experiments is shown the right side. Data shown are from at least two individual experiments with two or more mice of each genotype per experiment. Statistical significance was determined using a two-tailed student t-test, significant p-values are displayed as *<0.05, **<0.01.
